# Rationale and design of the TRIC‐I‐HF‐DZHK24 (TRICuspid Intervention in Heart Failure) trial

**DOI:** 10.1002/ejhf.3795

**Published:** 2025-08-11

**Authors:** Thomas J. Stocker, Tobias Geisler, Wolfgang Rottbauer, Edith Lubos, Christian Frerker, Niklas Schofer, Johanne Frank, Ralph Stephan von Bardeleben, Tobias Kister, Rico Osteresch, Alexander Lauten, Tienush Rassaf, Jürgen Rothe, David M. Leistner, Lukas Stolz, Victoria Kehl, Maria Grzeschniok, Andreas Hagendorff, Volker Rudolph, Stephan Baldus, Philipp Lurz, Steffen Massberg, Jörg Hausleiter, for the TRIC‐I‐HF‐DZHK24 Investigators, Jörg Hausleiter, Thomas J. Stocker, Steffen Massberg, Lukas Stolz, Michael Näbauer, Ludwig Weckbach, Philipp Doldi, Julia Novotny, Tobias Geisler, Meinrad Gawaz, Andreas Goldschmidt, Monika Zdanyte, Wolfgang Rottbauer, Mirjam Keßler, Edith Lubos, Christian Frerker, Roza Saraei, Ingo Eitel, Tobias Schmidt, Thomas Stiermaier, Momir Dejanovikj, Sebastian Kupp, Mulham Alhagi, Niklas Schofer, Daniel Kalbacher, Johanne Frank, Derk Frank, Matthias Lutz, Mohammed Saad, Stephan von Bardeleben, Philipp Lurz, Tobias Ruf, Theresa Gößler, Volker Rudolph, Kai Peter Friedrichs, Tanja Rudolph, Maria Ivannikova, Werner Scholtz, Hazem Omran, Tobias Kister, Holger Thiele, Rico Osteresch, Rainer Hambrecht, Alexander Lauten, Tienush Rassaf, Amir A. Mahabadi, Jürgen Rothe, Christian Besler, Mirjam Wild, Dirk Westermann, Nikolaus Löffelhardt, Roman Pfister, Stephan Baldus, Christos Iliadis, Dennis Mehrkens, Erion Xhepa, Michael Joner, Teresa Trenkwalder, Hector A. Covarrubias, Friederike Schürmann, Heribert Schunkert, David Leistner, Amin Polzin, Malte Kelm, Patrick Horn, Ralf Westenfeld, Eike Tigges, Georg Nickenig, Marcel Weber, Margarita Schulz, Johanna Vogelhuber, Dominik Nelles, Christian Butter, Mathias Konstandin, Norbert Frey, Philipp Nikolai, Raffi Bekeredjian, Alexander Wolf, Marc M. Vorpahl, Ulf Landmesser, Markus Reinthaler Fabian Barbieri, Joachim Schofer, Christina Brinkmann, Bernhard Unsöld, Kerstin Piayda, Sven Möbius‐Winkler, Christian Schulze

**Affiliations:** ^1^ Department of Cardiology, Department of Medicine I LMU University Hospital, LMU Munich Munich Germany; ^2^ German Center for Cardiovascular Research (DZHK), partner site Munich Heart Alliance Munich Germany; ^3^ Department of Cardiology and Angiology Eberhard Karls University Tübingen Tübingen Germany; ^4^ Department of Cardiology University Heart Center Ulm Germany; ^5^ Department of Internal Medicine and Cardiology, Katholisches Marienkrankenhaus Hamburg Germany; ^6^ University Heart Center, Schleswig‐Holstein University Lübeck Germany; ^7^ German Center for Cardiovascular Research (DZHK), partner site Hamburg‐Kiel‐Lübeck Lübeck Germany; ^8^ Department of Cardiology University Heart and Vascular Center Hamburg, University Medical Center Hamburg‐Eppendorf Hamburg Germany; ^9^ Department of Internal Medicine III, Cardiology, Angiologyand Intensive Care Medicine University Hospital Schleswig‐Holstein Kiel Kiel Germany; ^10^ Department of Cardiology Universitätsmedizin Johannes Gutenberg‐University Mainz Germany; ^11^ German Center for Cardiac and Vascular Research (DZHK), partner site Rhein‐Main; ^12^ Department of Cardiology Heart Center Leipzig at University of Leipzig Leipzig Germany; ^13^ Bremen Institute for Heart and Circulation Research (BIHKF) at the Klinikum Links der Weser Bremen Germany; ^14^ Department of General and Interventional Cardiology and Rhythmology Helios Klinikum Erfurt Erfurt Germany; ^15^ Department of Cardiology and Vascular Medicine West German Heart and Vascular Center, University Hospital Essen Essen Germany; ^16^ Division of Cardiology and Angiology University Heart Center Freiburg‐Bad Krozingen Bad Krozingen Germany; ^17^ Department of Cardiology and Angiology Goethe University Frankfurt am Main Germany; ^18^ Münchner Studienzentrum (MSZ), TUM School of Medicine and Health, Technical University of Munich Munich Germany; ^19^ Department of Cardiology Leipzig University Hospital Leipzig Germany; ^20^ General and Interventional Cardiology, Heart & Diabetes Center NRW Bad Oeynhausen Germany; ^21^ Department of Cardiology University of Cologne Cologne Germany

**Keywords:** Tricuspid regurgitation, Heart failure, Manifest right‐sided heart failure, Transcatheter tricuspid valve intervention, Tricuspid transcatheter edge‐to‐edge repair, Optimal medical therapy

## Abstract

**Aims:**

Tricuspid regurgitation (TR) is a detrimental disease frequently diagnosed in patients with right‐sided heart failure (HF). While transcatheter tricuspid valve interventions (TTVI) effectively reduce TR and improve quality of life (QoL) in earlier stages of the disease, their effect on reducing HF hospitalizations (HFH) and improving survival remains unclear.

**Methods:**

TRIC‐I‐HF‐DZHK24 (NCT04634266) is an investigator‐initiated, prospective, randomized, open‐label, multicentre strategy trial. Approximately 360 patients with severe TR and manifest right‐sided HF will be enrolled. In contrast to previous trials, subjects with increased risk for HFH will be selected as facilitated by specific inclusion criteria: HFH in the previous year, or presence of cardio‐renal syndrome, or evidence for cardio‐hepatic syndrome. Subjects will be randomized 2:1 to TTVI and optimal medical therapy (OMT) or continuation of OMT alone. All CE‐marked transcatheter repair devices including tricuspid transcatheter edge‐to‐edge repair (T‐TEER) or transcatheter tricuspid annuloplasty can be used for TTVI. The participating 29 study sites are highly experienced and treated a mean of 176 patients in 4.5 years with T‐TEER before study activation. The primary outcome will be assessed at 1 year. First, a composite of all‐cause mortality, HFH, and QoL improvement will be tested hierarchically. If positive, the combination of hard clinical endpoints including all‐cause mortality and HFH will be tested. Patients will be followed for a total of 3 years. The safety outcome comprises complications of TTVI, life‐threatening bleeding and death.

**Conclusions:**

The TRIC‐I‐HF‐DZHK24 trial will define the role of TTVI in patients with severe TR and right‐sided HF.

## Introduction

Tricuspid regurgitation (TR) is a highly prevalent disease independently associated with increased morbidity and mortality.^1^, ^2^ Medical treatment options are limited, and surgical results for isolated TR are discouraging in heart failure (HF) patients carrying increased operative risk.^3^, ^4^ While patients with severe TR and HF have been left without treatment options for many years, several transcatheter tricuspid valve interventions (TTVI) have been introduced in recent years. Tricuspid transcatheter edge‐to‐edge repair (T‐TEER) is the most frequently applied technique and received CE‐mark in the year 2020. Post‐market registries demonstrated high technical success rates and a very good safety profile of the available devices.^5^, ^6^ Propensity‐score matched prospective observational studies suggested a reduction of HF hospitalization (HFH) after TTVI in patients with significant right‐sided HF.^7^ Recent randomized controlled trials confirmed the safety of the technique and demonstrated significant improvement of quality of life (QoL) after T‐TEER, while HFH and mortality were unaffected.^8^, ^9^ However, both the TRILUMINATE Pivotal and Tri.Fr trials enrolled symptomatic patients but without additional selection criteria for right‐sided HF. Accordingly, the associated event rates for mortality and HFH within the trials were comparably low (1‐year mortality rate: 8.6% in TRILUMINATE Pivotal, and 3.4% in Tri.Fr).^8^, ^9^ Hence, the effect of TTVI in patients with manifest HF and underlying TR at advanced stages of the disease process remains unsolved. Therefore, the TRIC‐I‐HF‐DZHK24 (TRICuspid Intervention in Heart Failure) trial has been designed to assess the concept that TTVI will translate into a reduced morbidity and mortality in patients with severe TR and evidence for manifest right‐sided HF. TRIC‐I‐HF‐DZHK24 is an investigator‐initiated clinical strategy study and investigators may choose any suitable CE‐marked percutaneous system ‘on‐label’ for tricuspid valve repair. Transcatheter tricuspid repair devices for TTVI include T‐TEER and direct transcatheter tricuspid annuloplasty. Compared to the above mentioned previous clinical trials, the TRIC‐I‐HF‐DZHK24 study sites are considerably more experienced, and many sites accumulated T‐TEER experience before study initiation and even before devices received CE mark in 2020 by ‘off‐label’ MitraClip implantation. Patients will be randomly assigned in a 2:1 fashion to TTVI in addition to optimal medical therapy (OMT) (interventional group) or OMT alone (control group).

## Methods

### Study design and study aims

TRIC‐I‐HF‐DZHK24 is an investigator‐initiated, prospective, nationwide, multicentre, randomized, controlled, open‐label strategy trial in patients with TR and significant right‐sided HF. The trial is conducted in 29 German high‐volume heart valve centres and will assess whether OMT plus transcatheter tricuspid valve repair in HF patients with severe TR is more effective than OMT alone (*Graphical Abstract*). TRIC‐I‐HF‐DZHK24 will assess the strategy of TTVI using the individual optimal CE‐marked repair device. The optimal TTVI device for the individual patient is determined by the local heart team. TTVI repair devices studied in the trial include devices for T‐TEER and transcatheter tricuspid annuloplasty. Patients deemed for tricuspid valve replacement (orthotopic or heterotopic) are excluded from this trial. TRIC‐I‐HF‐DZHK24 will specifically enrol patients with signs of manifest HF to test the impact of TTVI on mortality and HFH in a vulnerable patient cohort with an increased risk for decompensation of HF. Previous randomized controlled trials enrolled patients without criteria for manifest right‐sided HF and observed comparably low event rates for HFH and mortality.

TRIC‐I‐HF‐DZHK24 is funded by the German Center for Cardiovascular Research (DZHK) and co‐funded by the university hospital of Ludwig‐Maximilians‐University, Munich, Germany, and Edwards Lifesciences. An executive steering committee composed of a group of experienced cardiologists in the treatment of HF and TR (J.H., V.R, R.S.V.B., P.L., S.B., S.M.) supports the design and conduct of the study. The industry sponsor is not involved in trial design or conduct and has no direct access to data during the course of the trial. A Data and Safety Monitoring Board will periodically review trial progress and evaluate the accumulated study data for participant safety. An independent Clinical Event Committee will independently adjudicate all hospitalizations. The trial is supported by the *Münchner Studienzentrum* (MSZ, TUM School of Medicine and Health in Munich) serving as central research organization. Statistical analysis will be conducted at MSZ (V.K.). The trial has been registered (NCT04634266) and is conducted in accordance with Good Clinical Practice, and ethical principles consistent with the Declaration of Helsinki. TRIC‐I‐HF‐DZHK24 has received approval from the responsible local ethics committees prior to inclusion of patients.

### Study cohort

#### Right‐sided heart failure and tricuspid regurgitation

Patients with right‐sided HF are clinically characterized by systemic fluid retention leading to peripheral oedema, ascites, anasarca, and hepatic distention. While TR reduces the effective right ventricular cardiac output, patients experience reduced physical activity, dyspnoea, and fatigue. The downstream consequences of right‐sided HF and severe TR include chronic kidney disease (cardio‐renal syndrome) and liver disease (cardio‐hepatic syndrome). Severity of TR is graded by transthoracic echocardiography (TTE) and transoesophageal echocardiography (TOE). Parameters and cut‐offs for the definition of severe TR are illustrated in *Table* *1*. The grading of TR should be performed in an euvolaemic state. In the event of congested HF, patients require diuretic therapy to achieve euvolaemia before grading of TR and assessment of trial eligibility. Different disease mechanisms can cause severe TR and the affected patients often suffer from various comorbidities. Primary TR is a less common cause for severe TR, however, prolapse and flail leaflets can be treated by transcatheter repair devices, and thus, patients with primary TR will be eligible for trial inclusion. Secondary TR is the most frequent cause of TR and can be further divided into ventricular secondary TR and atrial secondary TR. Causes of ventricular secondary TR include left ventricular disease (e.g. ischaemic cardiomyopathy, dilated cardiomyopathy, hypertensive HF, HF with preserved ejection fraction), pulmonary disease, and right ventricular dysfunction. Atrial secondary TR is often related to long‐standing atrial fibrillation and HF with preserved ejection fraction. Finally, transvalvular leads of cardiac implantable electronic devices (CIED) can cause severe TR and right‐sided HF. Given the variety in the aetiologies of TR, the trial population of TRIC‐I‐HF‐DZHK24 is expected to be heterogeneous in the underlying reasons for TR and right‐sided HF. Importantly, all patients enrolled into the trial should have significant TR identified as leading clinical problem that can be reasonably addressed by a strategy of transcatheter tricuspid repair.

**Table 1:** Echocardiographic grading of severe tricuspid regurgitation

	TR grade 3+ Severe	TR grade 4+ Massive	TR grade 5+ Torrential
Qualitative			
Color‐flow jet area	Large		
Flow convergence zone	Large throughout systole		
Semiquantitative			
Vena contracta, mm	7–13	14–20	>20
PISA radius, mm	>8 mm		
Hepatic vein flow	Systolic flow reversal		
Quantitative			
PISA EROA (cm^2^)	0.40–0.6	0.61–0.8	>80
Regurgitant volume (ml)	45–60	61–75	>75
New quantitative methods			
Regurgitant fraction (%)	>50		
3D Vena contracta (cm^2^)	0.75–0.95	0.96–1.15	>1.15

Modified after Hahn *et al*.^10^

3D, three‐dimensional; EROA, effective regurgitant orifice area; PISA, proximal isovelocity surface area; TR, tricuspid regurgitation.

#### Screening

Patients are screened for study eligibility by local TRIC‐I‐HF‐DZHK24 investigators of the respective study site without oversight of a central screening committee. Possible trial participants present at heart valve centres of TRIC‐I‐HF‐DZHK24 study sites after referral by general physicians or cardiologists for diagnostic and therapeutic evaluation of TR. In some cases, eligible patients may be identified after presentation with congested HF in the emergency departments of the respective study sites. In any case, patients require OMT for at least 30 days before enrolment into the trial. OMT includes diuretic treatment titrated to the individual optimal dose to reach euvolaemia. Patients are considered for trial participation if symptomatic and severe TR persists despite OMT.

#### Inclusion criteria

The TRIC‐I‐HF‐DZHK24 trial was designed to enrol patients with evidence for right‐sided HF and underlying severe TR. All patients are deemed at high risk for tricuspid surgery as evaluated by the local heart team. All patients have symptomatic TR as defined by New York Heart Association (NYHA) class II, III, or IV. Enrolled patients have severe or greater TR (TR grade 3+: severe; TR grade 4+: massive; TR grade 5+: torrential) as assessed by TTE and TOE (*Table* *1*).^10^ Patients should satisfy qualitative and (new) quantitative criteria for graduation of TR severity. The anatomy of the tricuspid valve needs to be treatable with repair devices of TTVI with high likelihood for optimal technical outcome as assessed by local experience. Additional inclusion criteria facilitate the selection of patients with manifest HF in advanced disease stages of TR carrying an increased risk for HFH. All patients must have a history of HFH during the previous 12 months, or show evidence for cardio‐renal syndrome, or cardio‐hepatic syndrome. One of the three circumstances needs to be fulfilled. Cardio‐renal syndrome is defined by the presence of chronic kidney disease (estimated glomerular filtration rate <60 ml/min) in combination with severe or greater TR and evidence for HF. Chronic kidney disease should be attributed in the context of HF for qualification as selection criteria. Patients with chronic kidney disease due to age, hypertension, or diabetes without evidence for HF should not be selected. Cardio‐hepatic syndrome is defined by severe or greater TR and elevation of two out of the three following laboratory parameters: bilirubin, alkaline phosphatase, or gamma glutamyl transferase.^11^ An elevation of bilirubin is defined by values above 1.1 mg/dl. The cut‐off values of alkaline phosphatase and gamma glutamyl transferase are set by the laboratory of the local study sites, since upper limits vary between gender, age, and laboratory calibration. All inclusion criteria are listed in *Table* *2*.

**Table 2:** Study inclusion and exclusion criteria

**Inclusion criteria** Subject is symptomatic due to severe TR despite OMT for at least 30 days based on judgment of the local heart team. Subject is at intermediate or greater estimated risk of morbidity and mortality, defined as: Hospitalization for heart failure during the previous 12 months, or Cardio‐renal syndrome^a^, or Cardio‐hepatic syndrome^b^. NYHA functional class II, III or IVa. Femoral vein access and tricuspid valve anatomy are determined to be feasible for transcatheter tricuspid repair (including sufficient image quality by TTE and TOE). Age ≥18 years at time of consent. Subject must provide written informed consent prior to any study‐related procedure. **Exclusion criteria** Presence of severe aortic, mitral or pulmonary valve disease. Surgical or interventional treatment of the aortic, mitral or pulmonary valves within 60 days prior to randomization. Excessive pulmonary hypertension (sPAP >70 mmHg) or substantial pre‐capillary pulmonary hypertension (mPAP >30 mmHg + TPG >17 mmHg, or mPAP >30 mmHg + PVR >5 WU) as assessed by right heart catheterization. Tricuspid valve stenosis (tricuspid mean gradient >5 mmHg). Pacemaker or ICD leads, only if transvalvular leads impede successful transcatheter tricuspid repair. Prior tricuspid valve procedures or unfavourable anatomy for successful transcatheter tricuspid repair (e.g. significant calcification, Ebstein anomaly, large coaptation defect). Chronic renal failure requiring dialysis. Insufficient image quality of tricuspid valve by TTE and TOE. Myocardial infarction or cerebrovascular accident within 90 days prior to randomization. Life expectancy of less than 12 months

ICD, implantable cardioverter‐defibrillator; mPAP, mean pulmonary arterial pressure; NYHA, New York Heart Association; OMT, optimal medical therapy; PVR, pulmonary vascular resistance; sPAP, systolic pulmonary arterial pressure; TOE, transoesophageal echocardiography; TPG, transpulmonary gradient; TR, tricuspid regurgitation; TTE, transthoracic echocardiography; WU, Wood unit.

^a^ Chronic kidney disease with an estimated glomerular filtration rate <60 ml/min in combination with severe or greater TR and evidence for heart failure.

^b^ Elevation of two out of the three following laboratory parameters: bilirubin, alkaline phosphatase, or gamma glutamyl transferase; in combination with severe or greater TR and evidence for heart failure.

#### Exclusion criteria

Several exclusion criteria were defined to warrant selection of patients that benefit most from TTVI. Patients with significant multivalvular disease are excluded. Accordingly, patients with surgical or interventional treatment of the aortic, mitral or pulmonary valves within 60 days prior to randomization cannot be enrolled. All patients receive TTE and TOE to understand the tricuspid valve anatomy, the right ventricular and chordal structure, the size of the coaptation gap, as well as the pathological mechanism and localization of TR. Based on the imaging evaluation, respective heart teams and interventional cardiologists should select patients with high likelihood for optimal technical results after TTVI. In this regard, patients with unfavourable tricuspid anatomies for TTVI are excluded (e.g. patients with large coaptation gaps unfavourable for leaflet‐ and annuloplasty‐based devices). Patients with transvalvular pacemaker or ICD leads can be included into the trial, however, if transvalvular leads significantly imped successful TTVI, patients should be excluded. Furthermore, patients with high risk of futility are excluded from the trial (e.g. patients with low life expectancy below 12 months). All patients require right heart catheterization before consideration of study participation to ensure comprehensive haemodynamic assessment of the study cohort. Patients with excessive pulmonary hypertension (invasive systolic pulmonary artery pressure >70 mmHg) are excluded. Furthermore, patients with significant pulmonary hypertension (invasive mean pulmonary artery pressure >30 mmHg) and substantial pre‐capillary contribution (transpulmonary gradient >17 mmHg or pulmonary vascular resistance >5 WU) are excluded. All exclusion criteria are listed in *Table* *2*. Number and information of screen‐failed patients (e.g. unfavourable anatomy for TTVI using a repair strategy) will be collected.

### Study procedures

#### Intervention scheme and trial flow

Heart failure patients with underlying severe TR are screened for eligibility (see Section Screening). If all inclusion criteria and none of the exclusion criteria are met (see Sections Inclusion criteria and Exclusion criteria) and the subject is willing to participate, informed consent for study participation is obtained. Further baseline examination is performed (see Section Data collection). The trial flow is illustrated in the *Graphical Abstract*. Randomization will be performed online using pre‐defined randomization lists with a 2:1 allocation ratio in favour of the interventional arm. Randomization is stratified by study centre and by TR grade. To assure balanced group sizes, a block‐wise randomization is applied. If a participant is randomized into the control arm, OMT is continued at discharge and follow‐up dates are scheduled. In the event of randomization into the interventional arm, TTVI should be scheduled as soon as possible within 14 days after randomization.

#### Optimal medical therapy

The OMT for the individual patient is defined by the treating physicians and the heart teams of the respective study sites. OMT in patients with chronic right‐sided HF includes diuretic therapy titrated to the optimal individual dosage. OMT stability is considered when adequate diuretic therapy at the maximum tolerated dosage is given for at least 30 days. In patients with HF and reduced ejection fraction, OMT includes guideline‐directed medical therapy as recommended by the guidelines for treatment of chronic heart failure.^12^

#### Transcatheter tricuspid valve intervention

Transcatheter tricuspid valve intervention should be scheduled as early as possible after randomization to the interventional arm to reduce the risk of adverse events before the intervention has been performed. The heart teams decide on the selection of the individual optimal repair device used for TTVI. For study participation, patients need to be treated with CE‐marked devices. At the time of study initiation, CE‐marked devices for TTVI included T‐TEER by PASCAL®, PASCAL ACE® (both Edwards Lifesciences) or TriClip® (Abbott Cardiovascular) as well as direct transcatheter tricuspid annuloplasty by CARDIOBAND® (Edwards Lifesciences). Orthotopic or heterotopic tricuspid valve replacement devices are not studied in this trial. Experience of study sites was set to a minimum of 20 TTVI cases for the desired device before treatment within the trial.

#### Cross‐over procedure

Cross‐over from the control arm to the interventional arm is possible if several circumstances are met. First, cross‐over procedures can be performed only after a minimum period of 30 days after randomization. Second, the consideration of cross‐over procedures is only possible in subjects that already met the primary endpoint for unplanned HFH. Third, patients required intravenous diuretic therapy for the treatment of congested HF as surrogate for the severity of the associated HFH event. After the cross‐over procedure, patients remain in the study and are scheduled for follow‐up assessments as originally planned.

### Data collection

#### Baseline evaluation

Diagnostic procedures required for study screening are performed as standard of care. This includes TTE, TOE, right heart catheterization and assessment of the NYHA functional class. If a subject is determined as eligible study candidate and informed consent was obtained, several baseline examinations need to be performed. This baseline evaluation includes information on demographics and past medical history, electrocardiogram (ECG), physical assessment, medication, laboratory results, and assessment of standardized 6‐min walking distance test. QoL is assessed by the standardized Kansas City Cardiomyopathy Questionnaire (KCCQ).

In collaboration with the DZHK, a predefined set of biosamples is taken and included into a central biobank, if the subject agrees and specific informed consent was obtained.

#### Echocardiographic image acquisition and central image analysis

A comprehensive TTE exam is required for screening and should be performed or repeated within 1 week (maximum of 4 weeks) before patient enrolment. All TTE images (screening TTE, discharge TTE in the event of randomization to the interventional arm, follow‐up TTEs after 1 month, 1, 2, and 3 years) are uploaded to a central image laboratory for further image assessment. Pseudonymization, image transfer, and image quality checks are performed by the respective core facilities at the DZHK. Additional TOE imaging is performed during the screening visit to evaluate the anatomy of the tricuspid valve and right heart. This anatomic information is required for clinical decision making and is used to assess eligibility for study participation. TOE images of the screening visit and TOE images of the TTVI procedure (in the event of randomization to the interventional arm) are uploaded to the central image laboratory.

#### Follow‐up assessment

Follow‐up evaluations are performed at 1 month (assessing procedural safety) and at 1, 2, and 3 years (assessing procedural efficacy) after randomization (control arm) or after discharge of TTVI procedure (interventional arm). All follow‐up evaluations should be performed on‐site and include assessment of NYHA functional class, TTE, ECG, physical assessment, medication, laboratory results, 6‐min walking distance test, and KCCQ. Events of hospitalization are specifically enquired, if not reported earlier. Adverse events and severe adverse events are documented immediately throughout the course of the study. Follow‐up TTEs are sent to the central image laboratory for independent analysis.

### Outcomes

#### Primary endpoint

The primary endpoint is composed by two endpoints that are tested hierarchically. The first primary endpoint is a hierarchical composite of all‐cause mortality, number of HFH, and lack of QoL improvement at 12 months. QoL is assessed by the KCCQ. If this first primary endpoint is statistically significant, a second primary endpoint of solely hard clinical endpoints will be tested. This second primary endpoint is a composite of time to all‐cause mortality or HFH, whichever occurs first, at a follow‐up of 12 months. HFH is defined by HF as the predominant reason for a hospital stay for more than 24 h based on signs and symptoms of HF that leads to the administration of intravenous, intensification of oral, or mechanical HF therapy. All hospitalizations will be reviewed and adjudicated regarding HF by an independent Clinical Event Committee.

#### Secondary endpoints

Secondary endpoints include all‐cause mortality, HFH (including frequency and length), change in QoL as assessed by KCCQ, re‐intervention for recurrent TR, change in NYHA functional class from baseline, change of 6‐min walking distance from baseline, change in echocardiographic parameters, change in peripheral oedema and subject weight, change of diuretic and HF medication from baseline, and change in laboratory markers for cardiac, renal and hepatic function. Sub‐group analyses are planned for different repair strategies. All endpoints are listed in *Table* *3*.

**Table 3:** Primary and secondary endpoints of the trial

**Hierarchical primary endpoint analysis** First primary endpoint: composite of all‐cause mortality, number of HFH, and lack of QoL improvement at 12 months. Second primary endpoint (tested only, if first primary endpoint is statistically significant): time to all‐cause mortality or HFH, whichever occurs first at 12 months.
**Secondary endpoints** All‐cause mortality Frequency and length of HFH Change in quality of life as assessed by KCCQ Re‐intervention rates for recurrent tricuspid regurgitation Change in NYHA class from baseline Change in 6‐min walk test distance from baseline Change in echocardiographic parameters (among others: TR grade, RV dimension and function, LV dimension and function, estimation of sPAP) Development of tricuspid stenosis (mean inflow gradient >5 mmHg) Change in peripheral oedema and subject weight Change in diuretic drugs and heart failure medications from baseline Change in laboratory markers for cardiac, renal and hepatic function
**Safety endpoint** Major adverse events related to the intervention during the first month, including mortality, life threatening bleeding (BARC classification type 3 and 5), major vascular or cardiac structural complications requiring intervention, or mechanical circulatory support.

BARC, Bleeding Academic Research Consortium; HFH, heart failure hospitalization; KCCQ, Kansas City Cardiomyopathy Questionnaire; LV, left ventricular; NYHA, New York Heart Association; QoL, quality of life; RV, right ventricular; sPAP, systolic pulmonary arterial pressure; TR, tricuspid regurgitation.

#### Safety endpoint

The safety endpoint is assessed at the 1‐month follow‐up visit and includes major adverse events related to the intervention and relevant for patients undergoing TTVI: mortality, life threatening bleeding (defined as Bleeding Academic Research Consortium bleeding type 3 and 5), major vascular or cardiac structural complications requiring intervention, or mechanical circulatory support.

### Statistical analysis

A sample size of 224 patients in the interventional arm and 112 patients in the control arm (total of 336 patients) are required to achieve 85% power assuming a win ratio of 1.818 and a significance level of 5% for a two‐sided test. This assumes the proportion of wins is 0.4, the proportion of losses is 0.22, and the proportion of ties is 0.38. Assuming a drop‐out rate of 10%, a total of 360 patients must be randomized, 240 patients in the interventional arm, and 120 patients in the control arm. All analyses will be performed on the full analysis set according to the intention‐to‐treat principle. Further, sensitivity analyses according to the per‐protocol set will also be performed.

The two primary endpoints will be analysed in a hierarchical testing procedure. The second primary endpoint will only be analysed, if the first primary endpoint delivers statistically significant results. The first primary endpoint will be analysed using the Finkelstein and Schoenfeld method, which calculates a win ratio based on pairwise comparisons between all possible patient pairs from the two treatment groups. In each pair, a ‘winner’ is determined by hierarchical comparison: first, based on time to all‐cause mortality; if both patients are alive or die at the same time, then based on the number of HFH; and if still tied, based on change in KCCQ score at 12 months. The win ratio is defined as the total number of wins in the treatment group divided by the total number of losses. The statistical significance of the win ratio will be evaluated at a two‐sided alpha level of 5%. The second primary endpoint, event‐free survival (composite of all‐cause mortality and HFH), will be evaluated using a two‐sided log‐rank test stratified by TR grading at a significance level of 5%, provided that the first primary endpoint shows a statistically significant result as per the hierarchical testing procedure. Kaplan–Meier analyses will be conducted for both treatment groups, and event‐free probabilities will be reported at 1, 2, and 3 years of follow‐up.

Secondary endpoints will be compared between the study arms using explorative statistics. The explorative significance level will be set to 5% and no adjustment will be made for multiple testing. Baseline and follow‐up demographic data will be described using standard descriptive statistics including frequency distributions, means and standard deviations, and medians and interquartile ranges. Cumulative incidence for all‐cause mortality and HFH will be calculated and presented as Kaplan–Meier time‐to‐event curves with comparison of the control and interventional arm.

## Discussion

The TRIC‐I‐HF‐DZHK24 trial is a multicentre, prospective, controlled, randomized, open‐label clinical study assessing the impact of TTVI on top of OMT in patients with HF due to underlying severe TR. T‐TEER is the best available, most abundantly used, and safest TTVI technique. Previous randomized controlled trials, namely the TRILUMINATE Pivotal and Tri.Fr trials, demonstrated a superiority of T‐TEER regarding QoL improvement, however, these trials could not identify an effect on all‐cause mortality and HFH.^8^, ^9^, ^13^ By specific inclusion criteria, the TRIC‐I‐HF‐DZHK24 trial will select for more advanced HF patients that are deemed at higher risk for HFH and will possibly benefit more from TTVI, than patients enrolled in TRILUMINATE Pivotal and Tri.Fr. The first primary endpoint is a composite of all‐cause mortality, HFH and patient‐centred QoL improvement. If this first primary endpoint is positive, a second primary endpoint, solely composed of hard clinical endpoints including all‐cause mortality and HFH at 1 year, will be tested hierarchically. Cross‐over from the control arm to the interventional arm is possible as important tool to enhance patient enrolment in a setting of commercially available TTVI devices, however, cross‐over procedures are only allowed by study protocol in the event of significant HFH with the necessity of intravenous diuretic therapy and after a blanking period of 1 month post‐randomization. Thus, the trial has the potential to bring definite evidence of TTVI on hard clinical endpoints in a high‐risk patient population.

Previous randomized controlled trials in the field of TTVI tested the impact of a specific device. In the TRILUMINATE Pivotal and Tri.Fr trials, only TriClip devices were implanted. In contrast, TRIC‐I‐HF‐DZHK24 is a strategic trial evaluating the impact of tricuspid valve repair by the utilization of commercially certified devices with CE mark. Another advantage of TRIC‐I‐HF‐DZHK24 in comparison to previous trials is the distinct TTVI experience of participating study sites, possibly resulting in improved procedural results with positive effect on study outcomes. Mean site experience in T‐TEER before local activation of the trial was very high: 176 ± 163 T‐TEER procedures, 54 ± 26 months of T‐TEER experience. Many sites collected experience in T‐TEER by off‐label implantation of MitraClip, before TriClip and PASCAL systems received CE mark in 2020.

Several methodological strengths in the design of the trial will improve the validity of the study results. First, echocardiographic data are independently reviewed by a central echo core lab to improve data quality of TTE and TOE image data including TR severity as well as right heart and left heart function. Second, a clinical event committee will adjudicate the correctness of HFH that is the most important component of the primary endpoint. Third, invasive haemodynamic assessment by right heart catheterization will be available in all patients of the trial since results of right heart catheterization are part of the in‐ and exclusion criteria. This facilitates comprehensive haemodynamic characterization of all patients enrolled into the trial.

### Limitations

The study has several limitations that warrant discussion. First, the study design will induce a selection bias with recruitment of patients at high‐volume sites. However, the experience of heart valve centres has been identified as significant contributor for clinical success and will improve the overall quality of the study.^6^ Second, the absence of a central screening committee might cause certain heterogeneity between study sites, however, eligibility for trial participation is defined by in‐ and exclusion criteria and is regularly monitored. Third, local investigators select eligible patients by identification of severe TR and clinical assessment of right‐sided HF. Patients with primarily left‐sided HF and neurohormonal activation can also develop fluid retention, peripheral oedema, and significant TR, and thus, might satisfy inclusion criteria for trial enrolment. Fourth, TRIC‐I‐HF‐DZHK24 selects patients with advanced right‐sided HF and increased risk for HFH due to specific selection criteria. The selection criteria ‘cardio‐renal syndrome’ might be influenced by concomitant diseases such as hypertensive or diabetic nephropathy. Fifth, the study requires multiple in‐hospital follow‐up visits of elderly patients with often reduced physical capacities. The risk of patients lost to follow‐up and incomplete data will be mitigated by multiple reminder strategies. Sixth, although mortality and HFH will be tested as hard clinical endpoints in TRIC‐I‐HF‐DZHK24, patient‐centred QoL outcomes are included in the first primary composite outcome by win‐ratio analysis of the KCCQ score. Finally, the lack of a sham procedure in the control group carries the risk of a placebo effect in the interventional arm with impact on patient‐centred QoL outcomes.

## Conclusion

TRIC‐I‐HF‐DZHK24 is a large, randomized clinical study to evaluate the strategic effect of TTVI plus OMT compared to OMT alone in HF patients with increased risk for HFH. The primary endpoint will be tested hierarchically. First, the hierarchical composite of all‐cause mortality, HFH, and QoL improvement will be tested. If positive, the second primary endpoint of hard clinical endpoints including all‐cause mortality and HFH will be tested. Patient recruitment started in March 2022 and ended in June 2025. The outcomes of the clinical study will provide clarification on whether TTVI in patients with significant TR and manifest right‐sided HF in addition to OMT is superior to a strategy of OMT alone.

## TRIC‐I‐HF‐DZHK24 Investigators

Prof. Dr. Jörg Hausleiter, Prof. Dr. Thomas J. Stocker, Prof. Dr. Steffen Massberg, Dr. Lukas Stolz, Prof. Dr. Michael Näbauer, Dr. Ludwig Weckbach, Dr. Philipp Doldi, Dr. Julia Novotny.

Department of Cardiology, Department of Medicine I, LMU University Hospital, LMU Munich, Munich, Germany.

German Center for Cardiovascular Research (DZHK), partner site Munich Heart Alliance, Munich, Germany.

Prof. Dr. Tobias Geisler, Prof. Dr. Meinrad Gawaz, Dr. Andreas Goldschmidt, Dr. Monika Zdanyte.

Department of Cardiology and Angiology, Eberhard Karls University Tübingen, Tübingen, Germany.

Prof. Dr. Wolfgang Rottbauer, Prof. Dr. Mirjam Keßler.

Department of Cardiology, University Heart Center, Ulm, Germany.

Prof. Dr. Edith Lubos.

Department of Internal Medicine and Cardiology, Katholisches Marienkrankenhaus, Hamburg, Germany.

Dr. Christian Frerker, Dr. Roza Saraei, Prof. Dr. Ingo Eitel, Dr. Tobias Schmidt, Prof. Dr. Thomas Stiermaier, Momir Dejanovikj, Sebastian Kupp, Mulham Alhagi.

University Heart Center, Schleswig‐Holstein University, Lübeck, Germany.

German Center for Cardiovascular Research (DZHK), partner site Hamburg‐Kiel‐Lübeck.

Dr. Niklas Schofer, Dr. Daniel Kalbacher.

Department of Cardiology, University Heart and Vascular Center Hamburg, University Medical Center Hamburg‐Eppendorf, Hamburg, Germany.

German Center for Cardiovascular Research (DZHK), partner site Hamburg‐Kiel‐Lübeck.

Dr. Johanne Frank, Prof. Dr. Derk Frank, Dr. Matthias Lutz, Dr. Mohammed Saad.

Department of Internal Medicine III, Cardiology, Angiology and Intensive Care Medicine, University Hospital Schleswig‐Holstein Kiel, Kiel, Germany.

German Center for Cardiovascular Research (DZHK), partner site Hamburg‐Kiel‐Lübeck.

Prof. Dr. Stephan von Bardeleben, Prof. Dr. Philipp Lurz, Dr. Tobias Ruf, Dr. Theresa Gößler.

Department of Cardiology, Universitätsmedizin Johannes Gutenberg‐University, Mainz, Germany.

German Center for Cardiac and Vascular Research (DZHK), partner site Rhein‐Main.

Prof. Dr. Volker Rudolph, Dr. Kai Peter Friedrichs, Prof. Dr. Tanja Rudolph, Maria Ivannikova, Dr. Werner Scholtz, Dr. Hazem Omran.

General and Interventional Cardiology, Heart & Diabetes Center NRW, Bad Oeynhausen, Germany.

Dr. Tobias Kister, Prof. Dr. Holger Thiele.

Department of Cardiology, Heart Center Leipzig at University of Leipzig, Leipzig, Germany.

Dr. Rico Osteresch, Prof. Dr. Rainer Hambrecht.

Bremen Institute for Heart and Circulation Research (BIHKF) at the Klinikum Links der Weser, Bremen, Germany.

Prof. Dr. Alexander Lauten.

Department of General and Interventional Cardiology and Rhythmology, Helios Klinikum Erfurt, German.

Prof. Dr. Tienush Rassaf, Prof. Dr. Amir A. Mahabadi.

Department of Cardiology and Vascular Medicine, West German Heart and Vascular Center, University Hospital Essen, Essen, Germany.

Dr. Jürgen Rothe, Dr. Christian Besler, Dr. Mirjam Wild, Prof. Dr. Dirk Westermann, Dr. Nikolaus Löffelhardt.

Division of Cardiology and Angiology, University Heart Center Freiburg‐Bad Krozingen, Bad Krozingen, Germany.

Prof. Dr. Roman Pfister, Prof. Dr. Stephan Baldus, Dr. Christos Iliadis, Dr. Dennis Mehrkens.

University of Cologne, Faculty of Medicine and University Hospital Cologne, Clinic III for Internal Medicine, Köln, Germany.

Dr. Erion Xhepa, Prof. Dr. Michael Joner, Dr. Teresa Trenkwalder, Dr. Hector A. Covarrubias, Dr. Friederike Schürmann, Prof. Dr. Heribert Schunkert.

Department of Cardiology, Deutsches Herzzentrum München, Munich, Germany.

German Center for Cardiovascular Research (DZHK), partner site Munich Heart Alliance, Munich, Germany.

Prof. Dr. David Leistner.

Department of Cardiology and Angiology, Goethe University, Frankfurt am Main, Germany.

German Center for Cardiac and Vascular Research (DZHK), partner site Rhein‐Main.

Prof. Dr. Amin Polzin, Prof. Dr. Malte Kelm, Dr. Patrick Horn, Dr. Ralf Westenfeld.

Department of Cardiology, Pulmonology, and Vascular Medicine, Heinrich Heine University Düsseldorf, Düsseldorf, Germany.

Dr. Eike Tigges.

Department of Cardiology and Intensive Care Medicine, Asklepios Clinic St. Georg, Hamburg, Germany.

German Center for Cardiovascular Research (DZHK), partner site Hamburg‐Kiel‐Lübeck.

Prof. Dr. Georg Nickenig, Dr. Marcel Weber, Dr. Margarita Schulz, Dr. Johanna Vogelhuber, Dr. Dominik Nelles.

Heart Center University Hospital Bonn, Bonn, Germany.

Prof. Dr. Christian Butter.

Department of Cardiology, University Hospital Heart Centre Brandenburg, Brandenburg Medical School (MHB) Theodor Fontane, Bernau/Neuruppin, Germany.

Prof. Dr. Mathias Konstandin, Prof. Dr. Norbert Frey.

Department of Cardiology, University Hospital Heidelberg, Ruprecht‐Karls‐University Heidelberg, Heidelberg, Germany.

German Center for Cardiovascular Research (DZHK), partner site Heidelberg/Mannheim, Heidelberg, Germany.

Dr. Philipp Nikolai, Prof. Dr. Raffi Bekeredjian.

Department of Cardiology, Robert‐Bosch‐Hospital, Stuttgart, Germany.

Dr. Alexander Wolf.

Department of Cardiology, School of Medicine, Witten/Herdecke University, Witten, Germany.

Prof. Dr. Marc M. Vorpahl.

Department of Cardiology, Helios Klinikum Siegburg, Germany.

Prof. Dr. Ulf Landmesser, Dr. Markus Reinthaler Dr. Fabian Barbieri.

Department of Cardiology, Angiology and Intensive Care Medicine, Deutsches Herzzentrum der Charité, Berlin, Germany.

German Center for Cardiovascular Research (DZHK), partner site Berlin, Berlin, Germany.

Prof. Dr. Joachim Schofer, Dr. Christina Brinkmann.

Department of Cardiology, Asklepios Clinic Sankt Georg, Hamburg, Germany.

Prof Dr. Bernhard Unsöld, Dr. Kerstin Piayda.

Medical Clinic I, Cardiology and Angiology, Justus‐Liebig‐University Giessen, Giessen, Germany.

Prof. Dr. Sven Möbius‐Winkler, Prof. Dr. Christian Schulze.

Department of Internal Medicine I, Cardiology, Angiology, Intensive Medical Care, University Hospital Jena, Jena, Germany.

Jörg Hausleiter, Thomas J. Stocker, Steffen Massberg, Lukas Stolz, Michael Näbauer, Ludwig Weckbach, Philipp Doldi, Julia Novotny, Tobias Geisler, Meinrad Gawaz, Andreas Goldschmidt, Monika Zdanyte, Wolfgang Rottbauer, Mirjam Keßler, Edith Lubos, Christian Frerker, Roza Saraei, Ingo Eitel, Tobias Schmidt, Thomas Stiermaier, Momir Dejanovikj, Sebastian Kupp, Mulham Alhagi, Niklas Schofer, Daniel Kalbacher, Johanne Frank, Derk Frank, Matthias Lutz, Mohammed Saad, Stephan von Bardeleben, Philipp Lurz, Tobias Ruf, Theresa Gößler., Volker Rudolph, Kai Peter Friedrichs, Tanja Rudolph, Maria Ivannikova, Werner Scholtz, Hazem Omran, Tobias Kister, Holger Thiele, Rico Osteresch, Rainer Hambrecht, Alexander Lauten, Tienush Rassaf, Amir A. Mahabadi, Jürgen Rothe, Christian Besler, Mirjam Wild, Dirk Westermann, Nikolaus Löffelhardt, Roman Pfister, Stephan Baldus, Christos Iliadis, Dennis Mehrkens, Erion Xhepa, Michael Joner, Teresa Trenkwalder, Hector A. Covarrubias, Friederike Schürmann, Heribert Schunkert, David Leistner, Amin Polzin, Malte Kelm, Patrick Horn, Ralf Westenfeld, Eike Tigges, Georg Nickenig, Marcel Weber, Margarita Schulz, Johanna Vogelhuber, Dominik Nelles, Christian Butter, Mathias Konstandin, Norbert Frey, Philipp Nikolai, Raffi Bekeredjian, Alexander Wolf, Marc M. Vorpahl, Ulf Landmesser, Markus Reinthaler Fabian Barbieri, Joachim Schofer, Christina Brinkmann, Bernhard Unsöld, Kerstin Piayda, Sven Möbius‐Winkler, Christian Schulze.

### Funding

The study is funded by the German Center for Cardiovascular Research (DZHK; website: https://dzhk.de/en) and co‐funded by the University Hospital of Ludwig‐Maximilians University Munich, Germany and Edwards Lifesciences.

**Conflict of interest**: T.J.S. received research support and speaker honoraria from Edwards Lifesciences. T.G. received research support, consultancy and speaker honoraria from Edwards Lifesciences and Medtronic and speaker honoraria from Abbott. C.F. received research support and speaker honoraria from Edwards Lifesciences and Abbott. N.S. received speaker honoraria and proctor fees from Edwards Lifesciences, as well as speaker honoraria from Abbott, Boston Scientific, Medtronic and HighLife SAS. T.R. received honoraria, lecture fees, and grant support from Edwards Lifesciences, AstraZeneca, Bayer, Novartis, BMS, Berlin Chemie, Daiicho‐Sankyo, Boehringer Ingelheim, Novo Nordisk, Cardiac Dimensions, and Pfizer, all unrelated to this work. D.M.L. received research support and speaker honoraria from Abbott Vascular and SMT, and received speaker honoraria from AstraZeneca, Abiomed, Bayer, Boston, Amgen, Daichi Novartis, Sankyo and Shockwave. L.S. received speaker honoraria from Edwards Lifesciences. V.R. received research support and consulting fees (to institution) by Abbott Vascular and Edwards Lifesciences. J.H. received research support and speaker honoraria from Edwards Lifesciences. All other authors have nothing to disclose.
